# The Abscess That Was Cancer: Pyogenic Liver Abscess as the Initial Manifestation of Hepatocellular Carcinoma in a Noncirrhotic Patient

**DOI:** 10.1155/crhe/9286315

**Published:** 2026-08-29

**Authors:** Francesco Siviero, Muhammad Ali Muslimani, Sonia Lerta, Sofia Frattola, Pietro Valsecchi, Raffaella Lissandrin, Laura Maiocchi, Marcello Maestri, Alessandro Vanoli, Angela Maria Di Matteo, Raffaele Bruno, Enrico Brunetti

**Affiliations:** ^1^ Department of Clinical, Surgical, Diagnostic and Pediatric Science, University of Pavia, Pavia, Italy, unipv.eu; ^2^ Division of Infectious and Tropical Diseases, IRCCS S. Matteo Hospital Foundation, Pavia, Italy; ^3^ Division of General Surgery 1, Department of Surgery, IRCCS S. Matteo Hospital Foundation, Pavia, Italy; ^4^ Department of Molecular Medicine, University of Pavia, Pavia, Italy, unipv.eu; ^5^ Division of Pathology, IRCCS S. Matteo Hospital Foundation, Pavia, Italy

**Keywords:** amoebic liver abscess, anchovy paste, biliary fistula, cognitive bias, Entamoeba, hepatocellular carcinoma, liver biopsy, noncirrhotic HCC, pyogenic liver abscess, tumor necrosis, ultrasound-guided percutaneous drainage, vancomycin-resistant *Enterococcus faecium*

## Abstract

**Background:**

Malignant and benign hepatic diseases may both present with systemic symptoms and necrotic liver cavities. Although rare and reported predominantly in East Asia, pyogenic liver abscess (PLA) can represent the initial manifestation of hepatocellular carcinoma (HCC), as necrotic tumors may mimic infectious abscesses.

**Case Presentation:**

A 63‐year‐old Italian man presented with a 2‐week history of fever and right upper quadrant pain. Computed tomography revealed a multiloculated lesion with irregular margins and a solid component in the right hepatic lobe, suggestive of a liver abscess, and empirical tigecycline therapy was initiated. Low‐titer *Entamoeba* spp. IgG antibodies were detected, and percutaneous aspiration yielded material initially mischaracterized as “anchovy paste,” raising suspicion for amoebic liver abscess. However, the patient failed to improve despite appropriate antiprotozoal therapy. Subsequent percutaneous drainage led to cavity resolution and symptom relief; all microbiological and parasitological tests were negative, and repeat *Entamoeba* serology became negative. Concurrent biopsy of the solid component revealed well‐differentiated pseudoglandular HCC. Magnetic resonance imaging demonstrated a biliary fistula associated with the neoplastic lesion, supporting a diagnosis of PLA secondary to HCC. The patient underwent right hepatectomy, complicated by intraoperative abscess rupture and postoperative intra‐abdominal abscesses caused by vancomycin‐resistant *Enterococcus faecium*.

**Discussion:**

PLA presenting as the initial manifestation of HCC is associated with poor prognosis, largely due to diagnostic delays. Limited clinical experience outside high‐prevalence regions and nonspecific clinical, laboratory, and imaging findings contribute to underrecognition of malignancy. Positive microbiological or serological results and transient clinical improvement during antimicrobial therapy do not exclude an underlying neoplasm. Liver biopsy and close radiologic follow‐up are essential in atypical or treatment‐refractory cases.

**Conclusion:**

PLA as a presenting feature of HCC is unusual, especially in Europe, yet poses substantial diagnostic and therapeutic challenges. Increased clinical awareness and early histopathological evaluation are crucial to avoid delayed oncologic diagnosis.

**Key points.**
• Pyogenic liver abscess can rarely represent the initial manifestation of hepatocellular carcinoma, even in the absence of previously documented cirrhosis.
• Clinical presentation, laboratory findings, and first‐line imaging are frequently nonspecific and may not distinguish hepatic abscesses from necrotic malignant lesions.
• Lack of clinical improvement despite appropriate antimicrobial therapy should prompt evaluation for underlying malignancy.
• Occult cirrhosis may be present despite the absence of imaging features, especially in patients with risk factors such as prior viral hepatitis and chronic alcohol exposure.
• Magnetic resonance imaging, including MR cholangiopancreatography, is crucial in detecting underlying neoplastic lesions and biliary complications that may contribute to abscess formation.
• Multidisciplinary management is essential due to the complex interaction between infection control and oncologic treatment strategies.

## 1. Introduction

Hepatocellular carcinoma (HCC) may occasionally manifest with an abscess‐like clinical presentation as a result of tumor necrosis driven by neoplasm‐associated granulocytosis [1]. Notably, the incidence of HCC presenting in noncirrhotic patients has been reported to reach up to 25% in certain cohorts [2].

Among benign necrotic hepatic lesions, pyogenic liver abscess (PLA) is the most frequent entity, typically arising secondary to hepatobiliary disease, intra‐abdominal infection, or portal venous translocation of enteric pathogens, particularly *Enterobacterales* [3]. Amoebic liver abscess (ALA) remains a major global cause of hepatic necrotic cavities; in European countries, its incidence has increased in recent years, largely owing to migration from endemic regions [4]. In contrast, fungal liver abscesses are uncommon and occur predominantly in severely immunocompromised hosts. Hepatic aspergillosis, in particular, is usually associated with hematologic malignancies, prolonged neutropenia, hematopoietic stem cell transplantation, intensive chemotherapy, and corticosteroid exposure [5].

Unlike bacterial and amoebic abscesses, which typically result from portal venous dissemination or infection with *Entamoeba histolytica*, hepatic *Aspergillus* involvement most often reflects hematogenous spread as part of invasive systemic disease [5]. These entities also differ substantially in their diagnostic and therapeutic implications. A recent systematic review reported that approximately 60% of the patients with *Aspergillus* liver abscesses received empirical antimicrobial therapy without antifungal coverage before the fungal etiology was established [6]. Furthermore, concomitant bacterial infection was documented in nearly one‐third of the cases, further complicating the differential diagnosis and therapeutic management [6].

The development of a liver abscess as the initial manifestation of HCC has been described predominantly in studies from East Asia, where the prevalence of both conditions is high [7–9]. An incidence of approximately 3.5% was reported in a large retrospective series [10]. Proposed pathogenic mechanisms include bacterial superinfection of necrotic tumor tissue and biliary obstruction caused by neoplastic thrombi [9]. However, limited knowledge of this presentation may lead to underrecognition of underlying malignancy during the acute infectious phase. Moreover, the absence of pre‐existing liver cirrhosis may reduce clinical suspicion and delay investigations aimed at identifying occult malignancy. This diagnostic challenge is compounded by the limited ability of first‐line imaging modalities to reliably distinguish PLA from ALA and, more rarely, from fungal hepatic abscesses.

We describe a case of HCC presenting as a multiloculated PLA and initially misdiagnosed as ALA, highlighting key diagnostic pitfalls and management challenges associated with this rare presentation. To our knowledge, this is the first report of PLA as the initial manifestation of HCC in Italy and, more broadly, in Western countries.

## 2. Case Presentation

A 63‐year‐old Italian man was admitted to our hospital with high‐grade fever unresponsive to empirical amoxicillin–clavulanate therapy, right upper abdominal pain radiating to the ipsilateral subscapular region and unintentional weight loss estimated at 6 kg. His medical history was notable for chronic alcohol abuse (up to 10 units/day) and a hepatitis C virus (HCV) infection treated 23 years earlier with interferon‐α plus ribavirin, with a documented sustained virologic response (SVR). On examination, isolated right hypochondrial pain with rebound tenderness was present. Laboratory investigations revealed leukocytosis with elevated acute‐phase reactants, including C‐reactive protein (27.04 mg/dL; reference < 0.5) and procalcitonin (0.98 μg/L; reference 0–0.5), together with mild hepatocellular and cholestatic enzyme abnormalities (alanine aminotransferase [ALT] 52 U/L; reference 11–34, aspartate aminotransferase [AST] 76 U/L; reference 11–39, total bilirubin 0.91 mg/dL; reference 0.2–1.1, and gamma‐glutamyl transferase [GGT] 133 U/L; reference 11–53), and mildly altered coagulation parameters. Serum alpha‐fetoprotein (AFP) was < 1.3 IU/mL (reference < 12).

**FIGURE 1:**
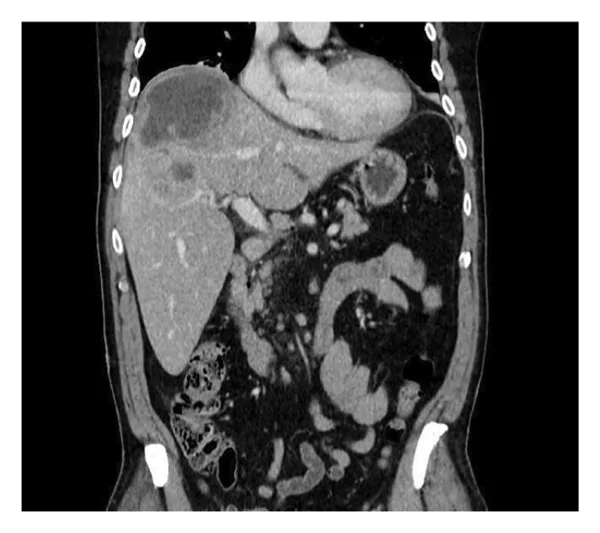
Multiloculated necrotic hepatic lesions demonstrated in the late portal phase on contrast‐enhanced abdominal CT.

Contrast‐enhanced abdominal computed tomography (CT) demonstrated a 12 × 9 × 11.5‐cm multilocular necrotic lesion with irregular margins involving hepatic segments VII and VIII. Rim enhancement in the late portal phase (Figure 1) suggested a hepatic abscess. Serum AFP and additional hepatobiliary tumor markers were within normal limits. Blood cultures were obtained, and empiric broad‐spectrum antimicrobial therapy with tigecycline was initiated following referral to the Infectious Diseases Department.

**FIGURE 2:**
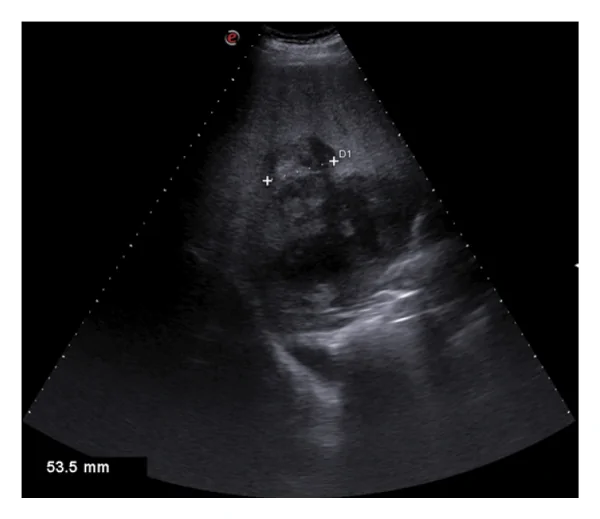
Heterogeneous, mixed‐echogenic hepatic cavities involving segments V and VII on ultrasonography.

Abdominal ultrasound confirmed multiple necrotic cavities, identifying two focal lesions of mixed echogenicity in segments V and VII (Figure 2). The former measured 5.3 mm and contained an isoechoic solid center, while the latter was hypoechoic with irregular, poorly defined margins. The remaining liver parenchyma appeared hyperechoic, consistent with steatosis, without evidence of cirrhosis.

**FIGURE 3:**
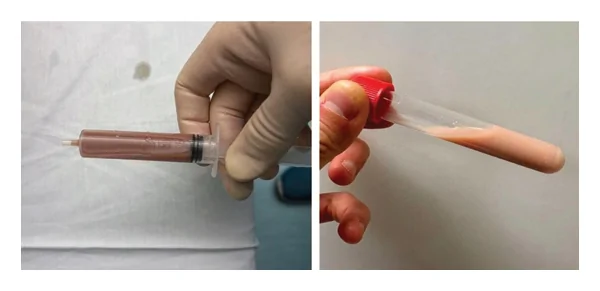
Aspirate obtained via US‐guided percutaneous drainage of a necrotic hepatic cavity.

Blood cultures collected prior to antibiotic initiation were negative. Serologic tests for *Brucella* spp., *Mycobacterium tuberculosis*, *Echinococcus* spp., and *Toxoplasma* spp. were all negative. Quantitative serology revealed low titers of anti‐*Entamoeba* IgG (1.4 [< 1.1 negative; > 1.1 positive]). Three days after antibiotic initiation, ultrasound‐guided percutaneous aspiration of the larger lesion was performed to avoid potential rupture given its size and proximity to the diaphragm. The aspirate yielded fluid that was mischaracterized as “anchovy paste,” particularly in light of positive *Entamoeba* serology (Figure 3). The patient experienced rapid resolution of abdominal pain. A single febrile episode with chills occurred postprocedure; repeated blood cultures were negative. Microscopic examination, dedicated Robinson culture, and Gal/GalNAc lectin antigen testing of the aspirate were all negative for *Entamoeba histolytica*. Bacterial cultures remained negative.

Given the imaging findings, serologic positivity, and gross appearance of the aspirated material, an initial diagnosis of ALA was made. The patient denied recent travel to endemic regions and reported no high‐risk sexual behavior. Metronidazole followed by paromomycin was administered, and tigecycline was replaced with ceftriaxone to cover possible bacterial coinfection. A total 2‐week antibiotic course was completed. Colonoscopy excluded intestinal amebiasis, and three serial stool examinations for ova and parasites were negative.

**FIGURE 4:**
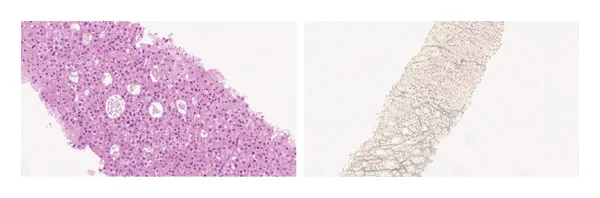
Well‐differentiated (G1) pseudoglandular hepatocellular carcinoma with associated loss of the reticulin framework on histopathological examination of liver biopsy tissue.

Despite therapy, the patient’s condition progressively worsened, with persistent hyperpyrexia and escalating right hypochondrial pain. Four weeks after the initial serologic test, repeat ELISA for *Entamoeba* spp. was negative, and repeat ultrasound showed no change in the lesions. A drainage catheter was inserted into the cavity, yielding sterile purulent brownish fluid, and a core biopsy of the solid lesion in segment V was obtained. Histopathological examination demonstrated foci of pseudoglandular, well‐differentiated (G1) HCC within areas of macrovesicular steatosis. Silver staining revealed partial reticulin framework loss (Figure 4).

**FIGURE 5:**
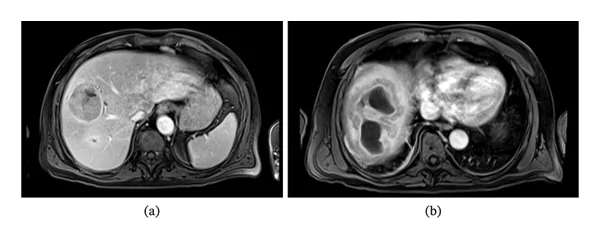
(a) Hepatic abscess containing a focal hepatocellular carcinoma component within segment V on T1‐weighted abdominal MRI. (b) Necrotic hepatic cavities involving segments VII and VIII on T1‐weighted abdominal MRI.

Magnetic resonance cholangiopancreatography (MRCP) further characterized the lesions. The segment V lesion was confirmed as an abscess containing a 1.1 × 1.1‐cm nodule with arterial‐phase enhancement and early washout (Figure 5a), radiologically consistent with HCC. A biliary fistula involving the right hepatic hemisystem and communicating with the necrotic cavity in segments VII and VIII was also identified (Figure 5b).

These findings supported the diagnosis of a PLA secondary to HCC. Moxifloxacin therapy was initiated because of suspected communication with the intestinal lumen. Over the ensuing 10 days, continuous drainage of necrotic material led to complete resolution of the cavities. All microbiological and parasitological tests remained negative. The patient became asymptomatic, with resolution of the fever. The drain was removed, and moxifloxacin was discontinued after a 2‐week course.

A multidisciplinary team—including specialists in infectious diseases, oncology, hepatobiliary surgery, and pathology—evaluated the case. The patient declined liver transplantation. Laparoscopic right lobectomy was, therefore, planned and performed 3 weeks after completion of antimicrobial therapy. Surveillance assays for carbapenem‐resistant Enterobacterales and methicillin‐resistant *Staphylococcus aureus* were negative. Prophylaxis with intravenous amoxicillin–clavulanate 2.4 g at time 0, 3 h, and 6 h postoperatively was administered.

Intraoperatively, a 5 × 4 × 4‐cm cavity containing yellow, pulpy material was identified in segments VII and VIII, with accidental spillage following abscess rupture. No cultures were obtained. A second firm, whitish, 6 × 4 × 4.5‐cm mass was found in segment V. Histopathological examination of intraoperatively obtained liver tissue confirmed pseudoglandular HCC with vascular invasion, necrosis, and chronic inflammatory changes. No microorganisms were microscopically identified. The surrounding liver parenchyma showed fibrotic septa delimiting nodules, consistent with micronodular cirrhosis—findings not evident on initial biopsy. A subsequent MRCP was successfully performed to close the biliary fistula via cannulation of the posterior right hepatic duct.

**FIGURE 6:**
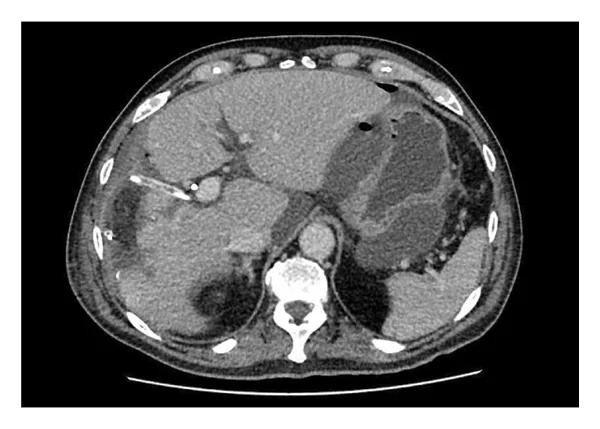
Postoperative appearance of multiloculated hepatic abscesses associated with *Enterococcus faecium* infection, with right hepatic ductal system stenting visualized during the portal phase of contrast‐enhanced abdominal CT.

Postoperative recovery was complicated by multilocular abdominal abscesses extending from the perihepatic to the perisplenic regions (Figure 6). Cultures from the drained collections grew vancomycin‐resistant *Enterococcus faecium* (VRE). Antibiotic treatment with linezolid and therapeutic percutaneous drainage of the purulent collections led to resolution of the lesions. The patient was subsequently discharged with scheduled close radiologic follow‐up.

## 3. Discussion

The clinical course of HCC presenting with abscess‐like features tends to be aggressive, likely reflecting extensive tumor necrosis, deep tissue invasion, and a higher rate of extrahepatic spread at the time of diagnosis [7–9]. This unfavorable prognosis is largely attributable to diagnostic delay, as infectious etiologies are typically prioritized during the initial evaluation of necrotic hepatic lesions.

Our case highlights several diagnostic pitfalls. Necrotic HCC may exhibit atypical radiologic features that closely resemble benign hepatic abscesses [11]. Moreover, first‐line imaging modalities—such as ultrasound and CT—demonstrate suboptimal sensitivity for detecting small or early neoplastic lesions in noncirrhotic livers [12], further contributing to potential misclassification during the acute infectious phase. Aspiration of necrotic cavities combined with tissue biopsy of any coexisting mass appears to be a critical step in identifying neoplastic tissue in cases of atypical or treatment‐refractory liver abscesses, as supported by previous reports [1, 7, 13]. While microbiological cultures of cavity fluid demonstrate good sensitivity for identifying bacterial pathogens when PLA coexists with HCC, the mere presence of a liver abscess does not exclude an underlying malignancy, and neither positive microbiological findings nor transient clinical improvement following antimicrobial therapy reliably rule out occult disease [1, 9]. In such scenarios, early tissue sampling may shorten diagnostic latency and potentially improve oncologic outcomes.

Our case further illustrates the risk of diagnostic anchoring—a well‐recognized cognitive bias—when serologic and macroscopic findings appear internally consistent with amoebic disease. The initial working diagnosis of ALA was reinforced by low‐titer *Entamoeba* seropositivity and the gross appearance of aspirated material interpreted as “anchovy paste,” creating a misleading sense of diagnostic coherence. However, liquefaction of necrotic hepatocytes can produce odorless, thick, brownish fluid that may be reminiscent of the classic appearance of amoebic abscesses. Similar findings have been reported in anaerobic PLA, further limiting the specificity of macroscopic assessment [14]. Additionally, serologic testing for *Entamoeba* spp. has limited specificity for active extraintestinal amebiasis, even in nonendemic regions [15]. False‐positive results have been described in association with bacterial, viral, and fungal infections, most commonly when assays rely on crude antigen preparations that promote nonspecific antibody binding [15, 16].

Integrating host‐related risk factors is essential. Chronic alcohol use and previous HCV infection may contribute to persistent hepatic injury and immune dysregulation, potentially predisposing to both infectious complications and HCC [7]. Classical risk factors for bacterial liver abscess include biliary tract disease, diabetes mellitus, intra‐abdominal infections, and underlying malignancy, the latter being independently associated with increased short‐term mortality [3, 17]. Hypervirulent *Klebsiella pneumoniae* strains deserve particular consideration because of their propensity to cause invasive liver abscess syndrome with metastatic dissemination [18]. ALA may occur in the absence of identifiable epidemiological risk factors, demanding diagnostic vigilance even in nonendemic settings [19]. By contrast, invasive hepatic aspergillosis predominantly affects the profoundly immunocompromised [5]; in these patients, negative bacterial cultures should prompt consideration of a fungal etiology, as conventional blood cultures and serum galactomannan have limited diagnostic sensitivity, frequently necessitating liver biopsy for definitive diagnosis [6, 20]. The absence of typical predisposing conditions in our patient, together with the atypical radiological evolution and failure to identify a convincing infectious source, ultimately reinforced the suspicion of an underlying malignant process rather than a primary bacterial, amoebic, or fungal liver abscess.

The occurrence of HCC in a patient without imaging evidence of cirrhosis is another noteworthy aspect. Although chronic hepatitis B or C infection, cirrhosis, and age ≥ 65 years are established risk factors for HCC‐associated PLA [7], increasing attention has been directed toward HCC arising in noncirrhotic livers. Current international guidelines do not recommend routine imaging surveillance following SVR in patients without cirrhosis due to the relatively low absolute risk of HCC [21–23]. Nevertheless, our findings emphasize the potential limitations of a surveillance strategy based solely on radiologic cirrhosis, particularly in patients with additional hepatic risk factors such as chronic alcohol exposure. Histologic evidence suggests that cirrhosis may precede radiologic detectability by months or years [24, 25], and early fibrotic remodeling may be recognized only incidentally on histopathological examination of bioptic samples. This observation supports a more individualized risk stratification approach in selected patients.

Serum tumor markers demonstrated limited diagnostic utility in this case. In noncirrhotic patients, serum AFP has limited utility as a screening tool, necessitating further diagnostic evaluation when HCC is suspected [12, 26]. Interestingly, elevated AFP and carbohydrate antigen 19–9 (CA 19–9) have been proposed as potentially specific markers of PLA associated with HCC in predominantly noncirrhotic cohorts [7] although these findings require further validation. Given that HCC arising in noncirrhotic livers is often diagnosed at advanced stages due to the absence of surveillance programs, liver biopsy may play a pivotal role in the early detection of neoplastic foci, potentially improving patient management and prognosis. Notably, a large Danish population‐based study reported a median age of 68 years among patients with PLA [27], underscoring that advanced age alone should not prompt biopsy in the absence of additional clinical or radiologic features suggestive of malignancy.

The limitations of liver biopsy must also be acknowledged. Aspiration or biopsy performed without prior suspicion of malignancy may result in tumor cell dissemination into the liver parenchyma or peritoneal cavity, potentially complicating subsequent management. Furthermore, biopsy may yield nondiagnostic samples because of extensive necrosis related to either the tumor or the infectious process [13]. Close radiologic follow‐up may, therefore, represent a valuable alternative or complementary strategy. Resolution of the necrotic cavity following antibiotic therapy and drainage may reveal previously obscured neoplastic foci, particularly when small or centrally necrotic. Second‐line imaging modalities may also be warranted despite higher costs and limited availability. Contrast‐enhanced MRI remains the preferred modality for detecting malignancy in noncirrhotic livers and provides superior characterization of biliary tract involvement [26]. In our patient, MRCP identified a biliary fistula connecting the necrotic cavity to the right hepatic ductal system, suggesting a potential pathogenic mechanism linking tumor progression and secondary infection. Although biliary fistulization is an uncommon complication of PLA, it represents a recognized pathway for persistent infection and often requires prolonged drainage and antimicrobial therapy [28]. Emerging imaging techniques, including spectral CT with quantitative iodine concentration analysis, have shown promise in differentiating necrotic HCC from PLA when conventional imaging findings are inconclusive. However, prospective validation is required before routine clinical implementation can be recommended [29].

Treatment of HCC complicated by PLA remains complex and requires a multidisciplinary approach. When technically feasible, surgical resection remains the preferred treatment for solitary HCC, particularly in noncirrhotic patients, as it offers the best potential for long‐term disease control [9, 26]. However, both infectious and neoplastic complications must be anticipated. Intraoperative rupture of an abscess, as observed in our case, is a rare but potentially fatal event associated with increased postoperative morbidity and mortality [30]. Tumor dissemination secondary to surgical manipulation has also been described [7]. Locoregional therapies, including transarterial chemoembolization, radiofrequency ablation, and hepatic artery ligation, may represent alternative strategies in selected patients with unresectable disease. The postoperative period remains particularly challenging because ischemic hepatic injury following surgical or locoregional treatment may facilitate anaerobic bacterial proliferation and exacerbate infection [9, 13]. Data regarding liver transplantation in patients presenting with PLA as the initial manifestation of HCC remain extremely limited. Concerns exist regarding the recurrence of infection due to post‐transplant immunosuppression. Therefore, transplantation should be cautiously considered and ideally deferred until complete eradication of infection has been achieved [31].

## 4. Conclusions

In high‐income countries, necrotic hepatic cavities are most commonly attributed to PLA or necrotic HCC. Nevertheless, PLA may rarely represent the initial manifestation of HCC, even outside high‐prevalence East Asian settings. This case provides several clinically relevant insights, including the potential for misleading serologic results, the limited specificity of aspirate macroscopic appearance, and the occurrence of HCC in patients without overt radiologic cirrhosis.

Accordingly, persistent, atypical, or treatment‐refractory liver abscesses should prompt early consideration of image‐guided aspiration combined with targeted liver biopsy to exclude occult neoplasia. Greater clinical awareness is essential to avoid diagnostic anchoring and therapeutic delay, particularly in low‐prevalence settings. Individualized risk stratification and a low threshold for histological assessment may improve diagnostic accuracy and patient outcomes in this challenging clinical scenario.

## Funding

There was no financial support, grant funding, or sponsorship received from any organization for the development and publication of this manuscript.

Open access publishing was facilitated by Universita di Pavia, as part of the Wiley ‐ CRUI‐CARE agreement.

## Disclosure

All authors are affiliated with the IRCCS S. Matteo Hospital Foundation of Pavia, where the patient was evaluated, treated, and followed up. The case was managed in accordance with institutional clinical protocols and standards of care.

## Ethics Statement

This case report was conducted in compliance with institutional ethical standards and the Declaration of Helsinki. Ethical approval was not required for this single‐patient case report as per the policies of the IRCCS S. Matteo Hospital Foundation of Pavia, provided that patient confidentiality is maintained.

## Consent

Written informed consent was obtained from the patient for publication of this case report, including the use of clinical data and imaging. All identifying information has been anonymized to protect patient privacy.

## Conflicts of Interest

The authors declare no conflicts of interest.

## Data Availability

The data that support the findings of this study are available from the corresponding author upon reasonable request.
